# arrayMap 2014: an updated cancer genome resource

**DOI:** 10.1093/nar/gku1123

**Published:** 2014-11-26

**Authors:** Haoyang Cai, Saumya Gupta, Prisni Rath, Ni Ai, Michael Baudis

**Affiliations:** 1 Institute of Molecular Life Sciences, University of Zurich, 8057 Zurich, Switzerland; 2 Swiss Institute of Bioinformatics, 8057 Zurich, Switzerland; 3 Center of Growth, Metabolism, and Aging, Key Laboratory of Bio-Resources and Eco-Environment, College of Life Sciences, Sichuan University, Chengdu 610064, Sichuan, China; 4 Centre for Integrative Genomics, University of Lausanne, 1015 Lausanne, Switzerland

## Abstract

Somatic copy number aberrations (CNA) represent a mutation type encountered in the majority of cancer genomes. Here, we present the 2014 edition of arrayMap (http://www.arraymap.org), a publicly accessible collection of pre-processed oncogenomic array data sets and CNA profiles, representing a vast range of human malignancies. Since the initial release, we have enhanced this resource both in content and especially with regard to data mining support. The 2014 release of arrayMap contains more than 64 000 genomic array data sets, representing about 250 tumor diagnoses. Data sets included in arrayMap have been assembled from public repositories as well as additional resources, and integrated by applying custom processing pipelines. Online tools have been upgraded for a more flexible array data visualization, including options for processing user provided, non-public data sets. Data integration has been improved by mapping to multiple editions of the human reference genome, with the majority of the data now being available for the UCSC hg18 as well as GRCh37 versions. The large amount of tumor CNA data in arrayMap can be freely downloaded by users to promote data mining projects, and to explore special events such as chromothripsis-like genome patterns.

## INTRODUCTION

Somatic genomic alterations refer to DNA sequence changes that are acquired during an individual's lifetime in the body's tissues (1,2). The type of unbalanced structural alterations which are called copy number aberrations/alterations (CNAs) are important subclasses of somatic DNA changes, including duplication, multi-copy amplification as well as homo- or heterozygous deletions of chromosomal segments (3). These frequently complex aberrations have been found in nearly all human tumor types, with regions spanning from several dozens of nucleotide bases to whole chromosomes (4–6). CNAs contribute to the initiation and progression of human malignancies by activating oncogenes, silencing tumor suppressor genes or disturbing gene expression through the involvement of regulatory elements (7,8). In the last two decades, array comparative genomic hybridization (aCGH) technologies have revolutionized cancer genome research by allowing the genome-wide detection of CNAs with high spatial resolution (9,10) (we use the term ‘aCGH’ both for dual color experiments as well as for single color oligonucleotide arrays that rely on external reference data sets).

The tens of thousands of tumor samples profiled by genomic arrays and deposited in public repositories allow researchers to identify patterns of non-random CNA events related to different cancer types, and to pinpoint involvement of specific cancer genes (6,11,12). A number of databases providing curated CNA data are available online, such as CaSNP (13), CanGEM (14) and Progenetix (15). These resources typically focus on particular data type, are derived from a restricted range of array platforms or do not contain probe-level data representation.

The public version of arrayMap was launched in 2012 (16) as a reference resource for array based genome data sets of copy number imbalances in human malignancies. It presents pre-processed cancer genome data, mainly derived from processed NCBI Gene Expression Omnibus (GEO) (17) and EBI ArrayExpress (18) data sets, but also including user provided and publication derived data, and provides online tools to perform basic data analysis and visualization. Users can freely download probe-level and segmented genomic array data from the web site. Typical uses of arrayMap data include investigation of potential markers for cancer diagnosis and therapy; identification of particular low incidence events (e.g. chromothripsis-like patterns) (19–21); large-scale data mining, such as construction of specific cancer type CNA patterns, and comparison of arrayMap data with users’ pre-publication data sets. Here, we summarize new developments in arrayMap content and utilities, which aim to increase data coverage and accuracy and importantly facilitate the use of this resource through a documented data interface.

## DATA CONTENT UPDATES

### Data growth

**Figure 1.**
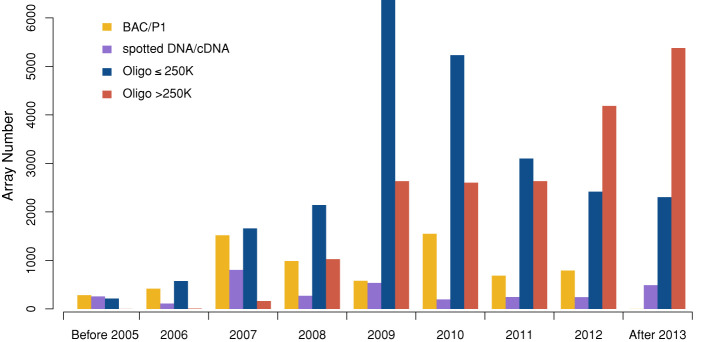
Distribution of arrays archived in arrayMap according to platform types and resolution. The usage of higher resolution platforms increased dramatically in recent years, with a concomitant decrease of especially low resolution BAC/P1 platforms.

At the time of its launch, the original arrayMap edition contained about 40 000 arrays from 260 different platforms, representing 224 cancer diagnoses as defined in the International Classification of Diseases in Oncology (ICD-O 3) (22). For the 2014 edition, the absolute number of data sets has been increased to 64 814 genomic copy number arrays from 985 experimental series, involving 343 array platforms. The primary data had been published in more than 700 original publications, and now represents 252 ICD-O cancer entities (Table 1). Over time, relatively low resolution array platforms are replaced by higher resolution or multi-function platforms. At the moment, the platform with the highest probe numbers in arrayMap contains about 2.2 million individual probes. In line with this trend, ∼60% of the added arrays contain more than 250K probes. Since the data generated by high resolution arrays increased rapidly in recent years, we anticipate that this growth trend will continue (Figure 1) with special impact on the detection of focal genomic imbalances.

**Table 1.** arrayMap content increase (as of 31 August 2014) compared to the initial release

Database content	Number of entries 2012	Number of entries 2014
Arrays	∼ 40 000	64 814
Series	533	985
Platforms	260	343
ICD-O cancer types	224	252
Publications	638	716
Patients	15 634	23 713

In this update, most novel samples were integrated from the NCBI GEO repository (17). Our main data selection criteria are that the data must be derived from human tumor samples and, where available, related germline DNA reference samples hybridized on single or dual color genomic array platforms. While primarily focusing on arrays with at least full autosomal coverage, we also integrated several studies with limited genome coverage which may provide useful information regarding gene specific CNAs in certain cancer types. In general, we used the formerly described pipeline (16) to re-process different data types. Briefly, for Affymetrix CEL files, we applied the aroma.affymetrix R package with the CRMAv.2 method (23) but utilized in-house scripts for data sets with available normalized probe intensity values. All probe signals were converted to log2 values, and Circular Binary Segmentation algorithm (24) was used for segmentation. For each array, empirical thresholds were assigned to call genomic gains and losses.

**Figure 2.**
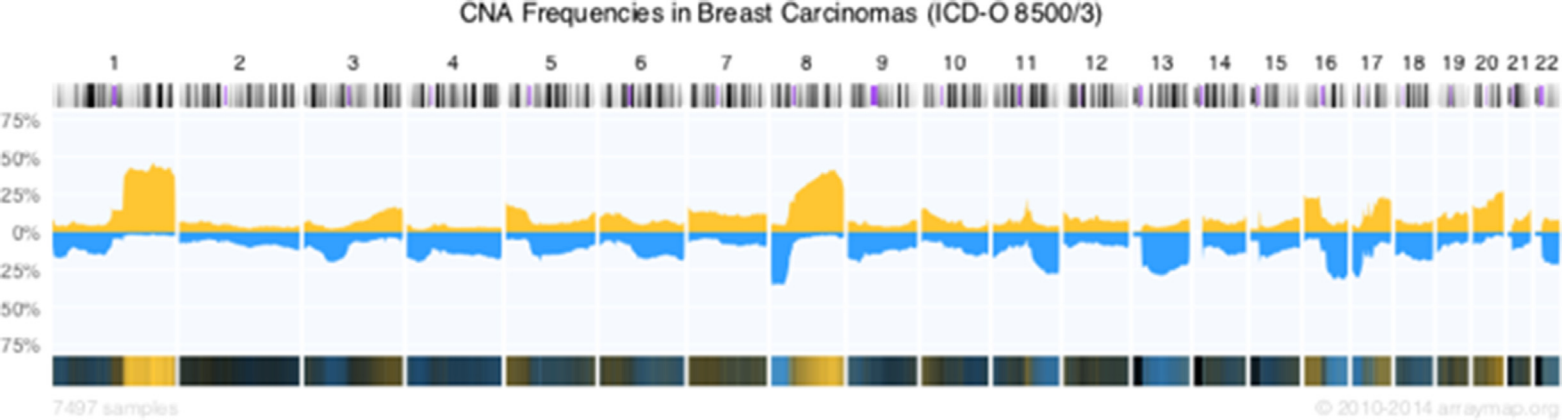
Frequency of copy number gains (up, yellow) and losses (down, blue), derived from 7497 breast cancer arrays (here limited to ICD 8500/3), as represented in the arrayMap database. For each of the included arrays, experiment specific CNA data can be accessed individually.

At the time of writing, data in arrayMap represent 252 ICD-O morphology codes. The largest of these are with 9551 samples ‘adenocarcinoma, NOS’ (8140/3; contains samples from e.g. prostate, gastric, colorectal and lung adenocarcinomas) and with 8188 samples ‘invasive carcinoma of no special type’ (8500/3; default histology for the majority of breast cancer samples; Figure 2). On the other end, 25 histologies are represented through a single array, among them e.g. ‘giant cell sarcoma’ and ‘islet cell carcinoma’. The complete list of ICD-O histologies is available through the supplements or can be accessed through the data API (application program interface; see below) at http://arraymap.org/api/?db=arraymap&api_out=icdmlist&icdm_m=0,8,9. Among the clinical entities, breast cancers constitute by far the largest category (8837 arrays) followed by non-small cell lung carcinomas (4112 arrays), acute myeloid leukemias (3641 arrays) and colorectal carcinomas (3047 arrays; Supplementary Table 2). The complete list is provided as supplement, or can be generated through calling ‘http://arraymap.org/api/?db=arraymap&api_out=cgrouplist&icdm_m=0,8,9’.

Compared to managed large-scale projects with frequent focus on a few predominant cancer types, the assembly of data from hundreds of individual studies has an inherent advantage in representing the heterogeneous landscape of human malignancies. As an example, when matching the arrayMap data to the content of the TCGA / ‘Pan-cancer project’ (25,26), one can observe that the 12 tumor types on which the ‘Pan-Cancer’ study has focused so far correspond to about half of the sample content in arrayMap (Table 2). While the efforts of the leading TCGA and ICGC (27) projects aim at a detailed multi-level description of molecular aberrations and their biological impact on cancer progression, the proportion of arrayMap samples from cancers not represented in those studies should serve as a reminder of the large number of ‘rare’ tumor types encountered in oncological practice, and the gap in our understanding of their molecular mechanisms. In our opinion, the arrayMap resource can prove especially useful in promoting oncogenomic data mining projects aimed at identifying exceptional tumor biologies.

**Table 2.** The proportions of TCGA ‘Pan-Cancer’ tumor diagnoses in arrayMap

Tumor types	Array numbers	Percentage
Breast carcinoma	8837	15.8%
Lung adenocarcinoma	3464	6.2%
Colon adenocarcinoma	3085	5.5%
Lymphoblastic acute myeloid leukemia	2848	5.1%
Glioblastoma	2517	4.5%
Ovarian carcinoma	2477	4.4%
Kidney renal clear-cell carcinoma	1473	2.6%
Head and neck squamous carcinoma	1423	2.5%
Uterine cervical and endometrial carcinoma	1013	1.8%
Lung squamous carcinoma	839	1.5%
Bladder carcinoma	689	1.2%
Rectal adenocarcinoma	250	0.4%
Other tumor types	27 101	48.5%

Besides the focus on cancer samples, the new edition of arrayMap also contains normal tissue samples that were used as controls in cancer profiling experiments. The amount of high resolution data from more than 8000 normal samples now allows for the creation of a matched number variation track, without relying on external resources (28–30) (Supplementary Figure S1). These data can be used to perform robust CNA data analysis, e.g. through providing a veto filter for the evaluation of focal (< 3–5 Mb) CNA events, which usually cannot be distinguished from germline variations without matched non-tumor samples.

### Genome reference assembly mapping

In the first release of arrayMap, all genomic mapping information for probe positions and derived CNA segments was converted to the human genome assembly UCSC hg18 (NCBI Build 36.1) (31, http://www.ncbi.nlm.nih.gov/projects/genome/assembly/grc/human/data/), to allow for the integration of the different platform types and experimental results. For this goal, a pipeline was generated to map the genomic positions for the thousands of array probes to the common ‘Golden Path’ edition. In recent years, new genome assemblies have been provided, (UCSC hg19 / GRCh37 and recently UCSC hg20 / GRCh38) with GRCh37 now frequently being used for referencing genomic array coordinates. When updating data from hg18 to newer assemblies, the change of probe coordinates may affect the composition of previously called CNA regions through un-mapping of some coordinates. To minimize this problem, for arrays with available probe values we first remapped all probe positions to GRCh37 using the UCSC Genome Browser's liftOver tool with intermediate BED files (30), and then re-segmented based on the derived probe positions. Although a few probes failed to be remapped during this procedure, the average remapping rate was as high as 99%. For a subset of e.g. literature derived data sets, segmentation data were processed directly. At the moment we are planning to migrate the database to the newest GRCh38 assembly.

## NEW AND ONGOING DEVELOPMENTS

### Web front end and data visualization

Some of the main strengths of the arraymap repository are the pre-computed visualization of some 10 000 probe-level genomic array data sets, as well as the graphical representation of CNA distributions based on curated clinical information, most notably the samples’ assignment to standardized diagnostic categories based on the WHO's ICD-O 3 schema (22). Since the arrayMap resource is based on the software framework developed for the Progenetix project (32), the data search and visualization updates reported in the 2014 Progenetix update (15) apply for the arrayMap resource, too. For the data selection, these include predefined aggregate data for ICD entities, tumor loci, SEER (33, http://www.seer.cancer.gov/popdata) categories as well as ‘clinical groups’, referring to samples with a common clinical context (e.g. ‘carcinomas: breast carcinomas’ including all types of epithelial breast tumors). Another option introduced with the latest Progenetix update and now applied to arrayMap is the geographic mapping of the included studies according. In the case of arrayMap, samples are mapped based on the submitting information from GEO, with a fallback to the corresponding author of the related publication. While this feature is not as useful as e.g. patient data derived origin mapping, it nevertheless offers a fast overview about enters with research activity in the corresponding cancer types and may support networking activities between research groups. Although the mapping information does not disclose the samples’ origins, the almost complete lack of data sets for large swaths of the globe (e.g. Africa, central Asia, South America) points to unmined cancer genome resources and paucity of research into possible epidemiological and environmental factors.

### API

The 2014 arrayMap release is the first to provide a RESTful data API. The API provides a variety of query and output parameters, with URL formatted (GET) requests returning server side processed data as JSON (Java Script object notation) objects, test/tabular data or images suitable for direct embedding or storage. A detailed and continuously updated documentation can be found online, in the arrayMap/Progenetix user guide at http://wiki.progenetix.org.

API Example 1: Sample data as JSON

The following query will return all samples from ICD-O 3 codes starting with ‘817’ (i.e. hepatocellular adenomas/carcinomas) from the arrayMap collection:

*http://arraymap.org/api/?db=arraymap&api_out=samples&api_doctype=json&icdm_m=817*

**Figure 3.**
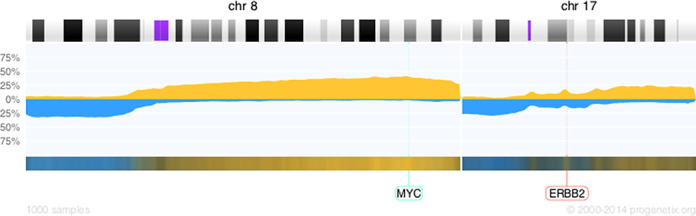
The histogram profile of breast cancer samples focusing on chromosomes 8 and 17 generated by the API, displaying the frequency of copy number gains (up, yellow) and losses (down, blue). Genomic map positions of two genes were included in the API call, and therefore are labeled at the bottom of the figure.

API Example 2:

The query will return a gain/loss frequency histogram for chromosomes 8 and 17, derived from 1000 random samples of ICD-O 8500/3 (breast carcinoma - ‘invasive carcinoma of no special type’), in the form of a PNG data stream (Figure 3):

*http://arraymap.org/api/?db=arraymap&markers_m=[MYC]8:128816862-128822853,[ERBB2]17:35104766-35138441&icdm_m=8500/3&randno=1000&api_out=histogram&chr2plot=8,17*

API Example 3:

The query will return the number of samples in arrayMap for ICD-O 8500/3, which have gain CNAs overlapping both the MYC and ERBB2 loci:

*http://arraymap.org/api/?db=arraymap&locus_m=8:128816862-128822853:1,17:35104766-35138441:1&icdm_m=8500/3&;api_out=count*

### API: R

With the ability to access the status matrix directly, one easily can import the data into an R data frame:

pgframe <- read.table(url(‘http://arraymap.org/api/?icdm_m=814&db=arraymap&api_out=matrix’), header = T, sep = ‘\t’, na = ‘NA’)

For the segment file, the same applies with ‘output=segments’:

segtable <- read.table(url(‘http://arraymap.org/api/?text_m=sezary&db=arraymap&api_out=segments’), header = T, sep = ‘\t’, na = ‘NA’)

To facilitate R integration of Progenetix/arrayMap data, we have developed a simple access function ‘pgDataLoader’ which can currently be accessed through GitHub (https://github.com/progenetix/pgRpi/). This publication's supplements include an example use case, describing the generation of gene specific Kaplan-Meyer survival plots from arrayMap data.

### User managed data

In this version of arrayMap, we provide some online support for the analysis and visualization of user private (i.e. pre-publication) array data sets. After registration by email, users are able to use on site storage facilities and recall previous performed analyses. For example, users can directly upload and visualize segmentation files, sample tables with ISCN karyotypes, or JSON files from a previous analysis. Data subsets from database queries can be reloaded and used for filtering and replotting. Additionally to these options, the analysis of raw / pre-processed probe data sets is supported in collaborative projects. Analysis input here can be e.g. Affymetrix genotyping array raw data (.CEL files), other platforms from log2 value lists, and pre-existing segmentation data.

## CONCLUSIONS AND FUTURE PERSPECTIVES

arrayMap is developed to provide a one stop resource of genomic copy number profiles of human tumors, as well as a series of online tools for meta-data analysis and mining. Although arrayMap is tightly integrated with and shows some content overlap with the Progenetix resource (http://www.progenetix.org), both data collections offer different scopes and data paradigms (Supplementary Figure S4). In contrast to arrayMap, which displays pre-processed but loosely evaluated experimental array data, Progenetix annotations are based on sample specific copy number data, from different technologies (chromosomal CGH, genomic arrays, genome sequencing), were the ‘called’ CNA had been either provided through a publication, or had been assessed from an active evaluation of the original experimental data. While the Progenetix resource has an advantage in providing genomic aberration data for an even wider diagnostic range than arrayMap (362 versus 252 ICD-O entities), it is more heterogeneous with respect to included technologies and spatial resolution of the CNA data sets (e.g. cytoband based cCGH data) which limits e.g. the detection of rare focal CNA events.

Since the launch of the resource in 2012, arrayMap underwent a number of quantitative, qualitative and functional improvements, most notably the increase in included data sets and scope of represented cancer entities, as well as the addition of programmatic access methods and Progenetix based selection and visualization updates. For the future expansion of the arrayMap resource, we are evaluating the additional inclusion of data sets from multi-functional platforms (e.g. methylation arrays, mutation-specific probe sets). Moreover, a robust platform agnostic quality rating system is under development, and will be integrated in our database. For the overall data set expansion, we intend to follow an incremental, dynamic update policy, with bi-annual reassessments of major data content and feature changes.

## SUPPLEMENTARY DATA

Supplementary Data are available at NAR Online.
